# Melatonin Attenuates Calcium Deposition from Vascular Smooth Muscle Cells by Activating Mitochondrial Fusion and Mitophagy via an AMPK/OPA1 Signaling Pathway

**DOI:** 10.1155/2020/5298483

**Published:** 2020-04-24

**Authors:** Wei Ren Chen, Yu Jie Zhou, Jia Qi Yang, Fang Liu, Xue Ping Wu, Yuan Sha

**Affiliations:** ^1^Department of Cardiology, Beijing Anzhen Hospital, Capital Medical University, Beijing Institute of Heart Lung and Blood Vessel Disease, Beijing Key Laboratory of Precision Medicine of Coronary Atherosclerotic Disease, Clinical Center for Coronary Heart Disease, Capital Medical University, Beijing, China; ^2^Department of Cardiology, Nanlou Division, Chinese PLA General Hospital at Beijing; National Clinical Research Center for Geriatric Diseases, China

## Abstract

Mitochondrial fusion/mitophagy plays a role in cardiovascular calcification. Melatonin has been shown to protect against cardiovascular disease. This study sought to explore whether melatonin attenuates vascular calcification by regulating mitochondrial fusion/mitophagy via the AMP-activated protein kinase/optic atrophy 1 (AMPK/OPA1) signaling pathway. The effects of melatonin on vascular calcification were investigated in vascular smooth muscle cells (VSMCs). Calcium deposits were visualized by Alizarin Red S staining, while calcium content and alkaline phosphatase (ALP) activity were used to evaluate osteogenic differentiation. Western blots were used to measure expression of runt-related transcription factor 2 (Runx2), mitofusin 2 (Mfn2), mito-light chain 3 (mito-LC3) II, and cleaved caspase 3. Melatonin markedly reduced calcium deposition and ALP activity. Runx2 and cleaved caspase 3 were downregulated in response to melatonin, whereas Mfn2 and mito-LC3II were enhanced and accompanied by decreased mitochondrial superoxide levels. Melatonin also maintained mitochondrial function and promoted mitochondrial fusion/mitophagy via the OPA1 pathway. However, OPA1 deletion abolished the protective effects of melatonin on VSMC calcification. Melatonin treatment significantly increased p-AMPK and OPA1 protein expression, whereas treatment with compound C ablated the observed benefits of melatonin treatment. Collectively, our results demonstrate that melatonin protects VSMCs against calcification by promoting mitochondrial fusion/mitophagy via the AMPK/OPA1 pathway.

## 1. Introduction

Vascular calcification (VC) is prevalent in coronary artery disease, and its extent predicts cardiovascular risk [1]. Causes of calcification in atherosclerosis include dysregulated matrix metabolism, epitaxial mineral deposition, inflammation, oxidative stress, and apoptosis [2]. VC is mainly mediated by vascular smooth muscle cells (VSMCs) [3] whose transformation from a contractile to osteogenic phenotype promotes the process of VC [4]. Osteoblastic differentiation of VSMCs is adjusted by the upregulation of several osteogenic genes, including runt-related transcription factor 2 (Runx2), alkaline phosphatase (ALP), and osteocalcin [5].

Mitochondrial fusion and mitophagy play a pivotal role in the development of VC [6, 7]. Optic atrophy 1 (OPA1) is a key regulator of mitochondrial fusion, and the AMP-activated protein kinase (AMPK)/OPA1 pathway is associated with mitochondrial fusion/mitophagy during cardiovascular disease [8, 9]. Phosphorylated-AMPK protein levels were shown to be decreased in VC, and ghrelin improved VC through AMPK activation [10]. Metformin was shown to inhibit beta-glycerophosphate- (*β*-GP-) induced impairment of mitochondrial biogenesis via AMPK activation in VC [6]. Melatonin, the main indoleamine produced by the pineal gland, is known to have anti-inflammatory, anticancer, and antioxidant activities [11]. Treatment with melatonin inhibited mitochondrial fission but promoted the fusion process in diabetic retina [12]. In human umbilical vein endothelial cells, melatonin inhibited calcium overload, dynamin-related protein1- (Drp1-) required mitochondrial fission, and mitochondrial apoptosis in response to lipopolysaccharide induction [13]. In addition, melatonin has been shown to attenuate the progression of atherosclerosis by inducing mitophagy and reducing inflammasome activation [14]. In our previous study, melatonin protected VSMCs against calcification by activating autophagy via the AMPK pathway [15]. Importantly, melatonin not only reduced calcium deposition and osteogenic differentiation but also suppressed apoptosis and inflammation [16]. Thus, the aim of the present study was to investigate whether melatonin reduces VSMC calcification by regulating mitochondrial fusion/mitophagy through the AMPK/OPA1 signaling pathway.

## 2. Materials and Methods

### 2.1. VSMC Isolation, Culture, and Calcification

VSMCs were isolated from the aortas of Sprague–Dawley rats (aged 4 weeks) using the explant method described in a previous study [17]. Immunohistochemical staining with anti-*α*-smooth muscle actin demonstrated these cells to be positive and indicated that the purity of VSMCs was more than 98%. For calcification, VSMCs were cultured with Dulbecco's Modified Eagle's Medium containing 10% fetal bovine serum and 10 mM *β*-GP for 14 days [18]. The medium was changed every 3 days. For drug treatment, melatonin was added before inducing calcification and continued for 14 days. To evaluate whether the AMPK pathway was involved in the protective effect of melatonin, VSMCs were treated with compound C (1 *μ*M, Sigma-Aldrich, St. Louis, MO) [10] for 14 days (*n* = 6 per group in one experiment).

### 2.2. Measurement of Calcium Deposition and ALP Activity

Alizarin Red S staining was performed to measure the formation of mineralized matrix (Gefan Biological Technology, Shanghai, China). Cells were decalcified with 0.6 mol/L HCl for 24 hours at 37°C, and the calcium contents were determined using a calcium colorimetric assay kit (Jiancheng Biological Engineering Institute, Nanjing, China). ALP activity was measured using an ALP kit (Beyotime Institute of Biotechnology, Shanghai, China).

### 2.3. Quantitative Real-Time Polymerase Chain Reaction (qRT-PCR)

Total RNA was extracted from VSMCs using a TRIzol reagent (Invitrogen, Carlsbad, CA) and then transcribed with a one-step RT-PCR kit (TransGen Biotech, Beijing, China) according to the manufacturer's instructions (Table 1). Quantification of gene expression was performed using an ABI PRISM 7500 Sequence Detection System (Applied Biosystems, Foster City, CA) with SYBR Green (TransGen Biotech). mRNA levels were determined by qRT-PCR in triplicate for each of the independently prepared RNAs and normalized to *β*-actin expression.

### 2.4. ELISA

Monocyte chemotactic protein (MCP-1), tumor necrosis factor *α* (TNF*α*), and interleukin-6 (IL-6) levels were analyzed by sandwich ELISA (BioCheck, Foster City, CA). In addition, a glutathione peroxidase (GPx) assay kit, glutathione (GSH) assay kit, and superoxide dismutase (SOD) assay kit were used to evaluate GPx, GSH, and SOD levels, respectively. Caspase 3 activity was monitored using a caspase 3 assay kit (Beyotime Institute of Biotechnology). A glucose uptake assay kit was used to measure glucose uptake (Abnova, Taiwan, China; KA4086), while a lactate assay kit was used to determine concentrations of lactate (Abcam, Cambridge, UK; ab65331).

### 2.5. MitoSOX Red Mitochondrial Superoxide Indicator, Mitochondrial Membrane Potential, Mitochondrial Permeability Transition Pore (mPTP) Opening, Mitochondrial Morphology, and Mitochondrial Respiratory Assays

Mitochondrial superoxide production was detected using a MitoSOX Red mitochondrial superoxide indicator (YESEN, Shanghai, China). A JC-1 Kit was used to evaluate mitochondrial membrane potential (Beyotime Institute of Biotechnology). Opening of the mPTP was measured as the rapid dissipation of tetramethylrhodamine ethyl ester fluorescence. Arbitrary mPTP opening time was assessed as the time to the loss of average tetramethylrhodamine ethyl ester fluorescence intensity by half between the initial and residual fluorescence intensity. Cellular ATP levels were determined using an ATP Assay Kit (Beyotime Institute of Biotechnology). Mitochondrial morphology was assessed using MitoTracker Red images in conjunction with NIH ImageJ software (http://imagej.nih.gov). Mitochondrial State 3 or State 4 respiration was achieved by adding ADP according to the general method described previously [19].

### 2.6. Western Blots

Following experimental treatment, VSMCs were lysed with RIPA lysis buffer containing protease inhibitor (Beyotime Institute of Biotechnology) for 30 minutes and centrifuged at 14,000 × g for 30 minutes. A bicinchoninic acid protein estimation kit was used to evaluate protein concentrations (Beyotime Institute of Biotechnology). Equal amounts of protein (50 *μ*g) were loaded into the wells of a 10% sodium dodecyl sulfate-polyacrylamide gel. Proteins were separated by gel electrophoresis and transferred to a polyvinylidene difluoride membrane (Millipore, Burlington, MA). Membranes were blocked with 5% milk in Tris-buffered saline containing 0.05% Tween 20 (TBST) at room temperature for 1 hour followed by overnight incubation at 4°C with the following primary antibodies: anti-Runx2 (1 : 1000, Abcam, ab76956), anti-OPA1 (1 : 1000, Abcam, ab42364), anti-cleaved caspase 3 (1 : 1000, Abcam, ab13847), anti-mitochondrial fission protein 1 (Fis1; 1 : 1000, Abcam, ab71498), anti-mitofusin 2 (Mfn2; 1 : 1000, Abcam, ab56889), anti-light chain 3 II/I (LC3II/I; 1 : 1000, Cell Signaling Technology, Danvers, MA; 3868/4599), anti-beclin1 (1 : 1000, Abcam, ab62557), anti-AMPK (1 : 1000, Abcam, ab131512), anti-p-AMPK (1 : 1000, Abcam, ab23875), and anti-*β*-actin (1 : 1000, Abcam, ab8227). After overnight incubation, membranes were washed with TBST and further incubated with an appropriate secondary antibody at room temperature for 60 minutes. Membranes were developed with an enhanced chemiluminescence reagent.

### 2.7. Immunofluorescence and TUNEL Method

For immunofluorescence assays, cells were fixed with 4% paraformaldehyde for 30 minutes, followed by permeabilization using 0.5% Triton X-100 for 10 minutes. Next, cells were blocked with 5% bovine serum albumin for 1 hour and incubated with primary antibodies against Runx2 (1 : 200, Cell Signaling Technology), OPA1 (1 : 200, Cell Signaling Technology), cleaved caspase 3 (1 : 200, Cell Signaling Technology), LC3II (1 : 200, Cell Signaling Technology), or p-AMPK (1 : 200, Cell Signaling Technology) overnight at 4°C. The next day, cells were incubated with an appropriate secondary antibody (1 : 200, Cell Signaling Technology) for 1 hour at 37°C. Images were acquired using a fluorescence microscope (Olympus DX51, Tokyo, Japan). Apoptosis was detected using a terminal deoxynucleotidyl transferase dUTP nick end labeling (TUNEL) assay (Roche, Basel, Switzerland) according to the manufacturer's instructions. The apoptosis index was calculated by calculating the percentage of TUNEL-positive cells to total nucleated cells stained by DAPI.

### 2.8. Transfection

Scrambled siRNA against OPA1 or autophagy protein 5 (ATG5) were purchased from Santa Cruz Biotechnology (Dallas, TX). For RNAi knockdown, cells were seeded in plates containing medium without antibiotics for 24 hours before transfection. siRNAs were transfected into cells using Lipofectamine 2000 (Invitrogen) in serum-free Opti-MEM (Invitrogen), according to the manufacturer's instructions. Recombinant OPA1 adenovirus (Ad-OPA1) was purchased from Cyagen Company (Sunnyvale, CA).

## 3. Statistical Analysis

Data are described as the mean ± standard deviation (SD) and were analyzed by one-way analysis of variance followed by Tukey's test. The limit of statistical significance between treatment and control groups was *P* < 0.05.

## 4. Results

### 4.1. Melatonin Attenuated *β*-GP-Induced VSMC Calcification through the OPA1 Pathway

**Figure 1:**
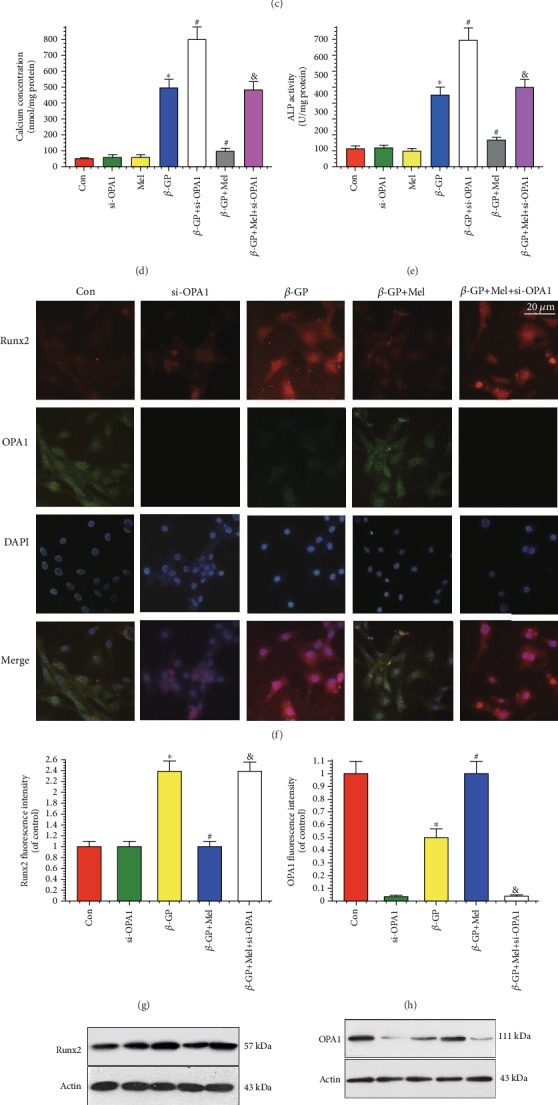
Melatonin reduced *β*-GP-induced calcium deposition via OPA1 in VSMCs (*n* = 6/group). VSMCs were cultured with Dulbecco's Modified Eagle's Medium containing 10% fetal bovine serum and 10 mM *β*-GP for 14 days. (a, b) Results of different concentrations of melatonin on calcium content and alkaline phosphatase (ALP) activity. (c) Result of melatonin (5 *μ*M) on Alizarin Red S staining. (d) Result of calcium concentration. (e) Result of ALP activity. (f–h) Result of immunofluorescence assay (red signal represents Runx2, green signal represents OPA1). (i, j) Results of Runx2 and OPA1 protein expression. ^∗^*P* < 0.05 vs. Con, ^#^*P* < 0.05 vs. *β*-GP, and ^&^*P* < 0.05 vs. *β*-GP+Mel.

As shown in Figures 1(a) and 1(b), 5 *μ*M of melatonin significantly reduced calcium content and decreased ALP activity in calcifying VSMCs. Therefore, most experiments were performed using a melatonin concentration of 5 *μ*M. Alizarin Red S staining indicated that *β*-GP promoted the calcification of VSMCs, while melatonin significantly inhibited *β*-GP-induced calcification (Figure 1(c)). Calcium content in the melatonin group (107 ± 13) was decreased compared with the control group (502 ± 55) (*P* < 0.05) (Figure 1(d)). Moreover, ALP activity was significantly increased in response to *β*-GP, while melatonin significantly reduced ALP activity (151 ± 11 in melatonin vs. 406 ± 42 in *β*-GP, *P* < 0.05) (Figure 1(e)). However, OPA1 deletion reduced the protective effects of melatonin on VSMC calcification.

An immunofluorescence assay was used to evaluate Runx2 and OPA1 expression in VSMCs. Runx2 protein expression was increased in the *β*-GP group but decreased in the *β*-GP and melatonin cotreatment (*β*-GP+melatonin) group. We also found that *β*-GP decreased OPA1 expression, while melatonin treatment significantly upregulated OPA1 expression; however, OPA1 knockout reversed this phenomenon (Figures 1(f)–1(h)). Western blot results showed that Runx2 and OPA1 expression levels were similar to those shown in Figure 1(f) amongst the control, *β*-GP, and *β*-GP+melatonin groups (Figures 1(i) and 1(j)). Overall, these results show that melatonin protected VSMCs against calcification by promoting OPA1 expression.

### 4.2. Melatonin Reduced VSMC Inflammatory Response and Oxidative Stress through the OPA1 Pathway

**Figure 2:**
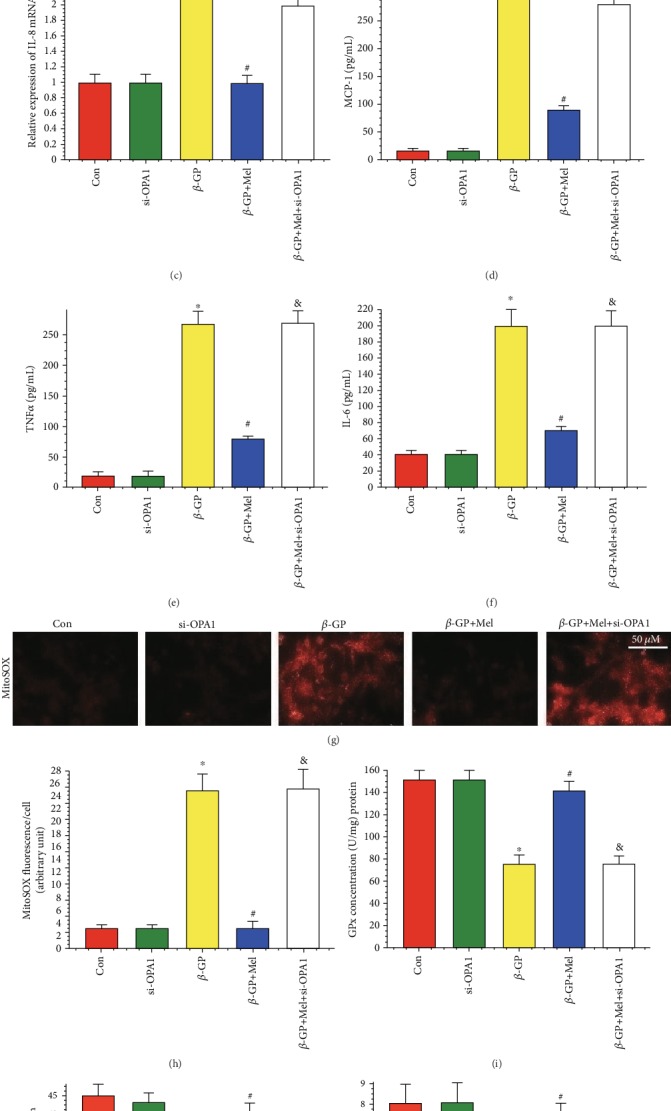
Effects of melatonin on inflammatory response and oxidative stress in *β*-GP-treated VSMCs (*n* = 6/group). (a) Result of matrix metalloprotein 9 (MMP9) mRNA expression. (b) Result of macrophage inhibitory protein-1*α* (MIP1*α*) mRNA expression. (c) Result of interleukin-8 (IL-8) mRNA expression. (d) Result of monocyte chemotactic protein (MCP-1) level. (e) Result of tumor necrosis factor *α* (TNF*α*) level. (f) Result of interleukin-6 (IL-6) level. (g, h) MitoSOX for mitochondrial superoxide formation. (i–k) Results of glutathione peroxidase (GPx), L-glutathione (GSH), and superoxide dismutase (SOD) levels. ^∗^*P* < 0.05 vs. Con, ^#^*P* < 0.05 vs. *β*-GP, and ^&^*P* < 0.05 vs. *β*-GP+Mel.

qRT-PCR analysis showed that mRNA expression of inflammatory factors was increased in the *β*-GP group and decreased in the *β*-GP+melatonin group. However, deletion of OPA1 significantly increased these levels, despite treatment with melatonin (Figures 2(a)–2(c)). ELISA results further confirmed this finding. Melatonin significantly reduced levels of inflammatory factors in *β*-GP-induced calcified VSMCs, and the loss of OPA1 nullified the effect of melatonin on VSMC inflammation (Figures 2(d)–2(f)).

To investigate the relationship between melatonin-mediated vascular protection and oxidative stress, we measured levels of mitochondrial superoxide in VSMCs. *β*-GP increased levels of mitochondrial superoxide, while melatonin reduced these levels through OPA1 regulation (Figures 2(g) and 2(h)). Moreover, melatonin promoted the levels of antioxidant compounds (GPx, GSH, and SOD), while OPA1 ablation may inhibit these effects (Figures 2(i)–2(k)). Taken together, these results suggest that melatonin could reduce inflammation and oxidative stress via OPA1 regulation in calcifying VSMCs.

### 4.3. Melatonin Protected VSMCs against Apoptosis through the OPA1 Pathway

**Figure 3:**
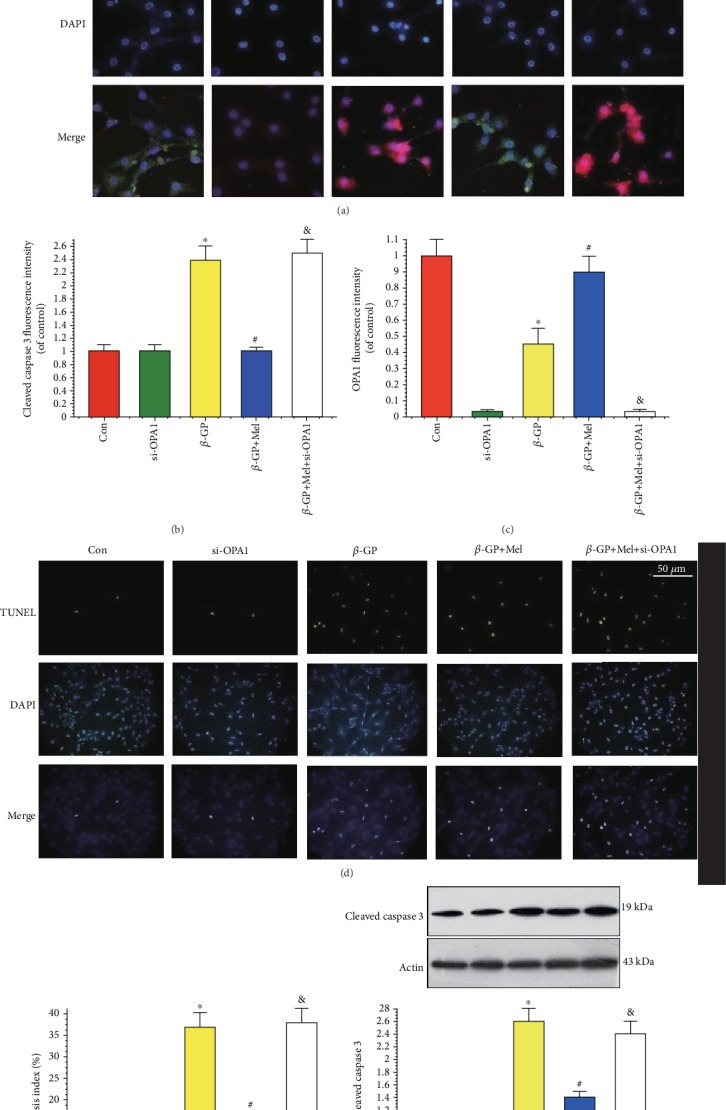
Effects of melatonin on the apoptosis in *β*-GP-treated VSMCs (*n* = 6/group). (a–c) Confocal microscopy of immunofluorescence staining of cleaved caspase 3 (red) and OPA1 (green). (d, e) The apoptosis of VSMC was determined by TUNEL staining. (f) Results of cleaved caspase 3 expression. ^∗^*P* < 0.05 vs. Con, ^#^*P* < 0.05 vs. *β*-GP, and ^&^*P* < 0.05 vs. *β*-GP+Mel.

Immunofluorescence staining results showed that cleaved caspase 3 was increased in the *β*-GP group, but reduced in the *β*-GP+melatonin group. However, the loss of OPA1 significantly promoted cleaved caspase 3 expression compared with the melatonin group (Figures 3(a)–3(c)).

A TUNEL assay was used to evaluate the incidence of apoptosis in VSMCs. Compared with the *β*-GP group, melatonin treatment significantly inhibited apoptosis in VSMCs (Figures 3(d) and 3(e)). In addition, cleaved caspase 3 protein expression was decreased in the melatonin group (Figure 3(f)). However, OPA1 deficiency abolished the protective effects of melatonin on VSMC apoptosis. Thus, these results indicate that melatonin protected VSMCs against apoptosis via the OPA1 pathway.

### 4.4. Melatonin Maintained Mitochondrial Function through the OPA1 Pathway

**Figure 4:**
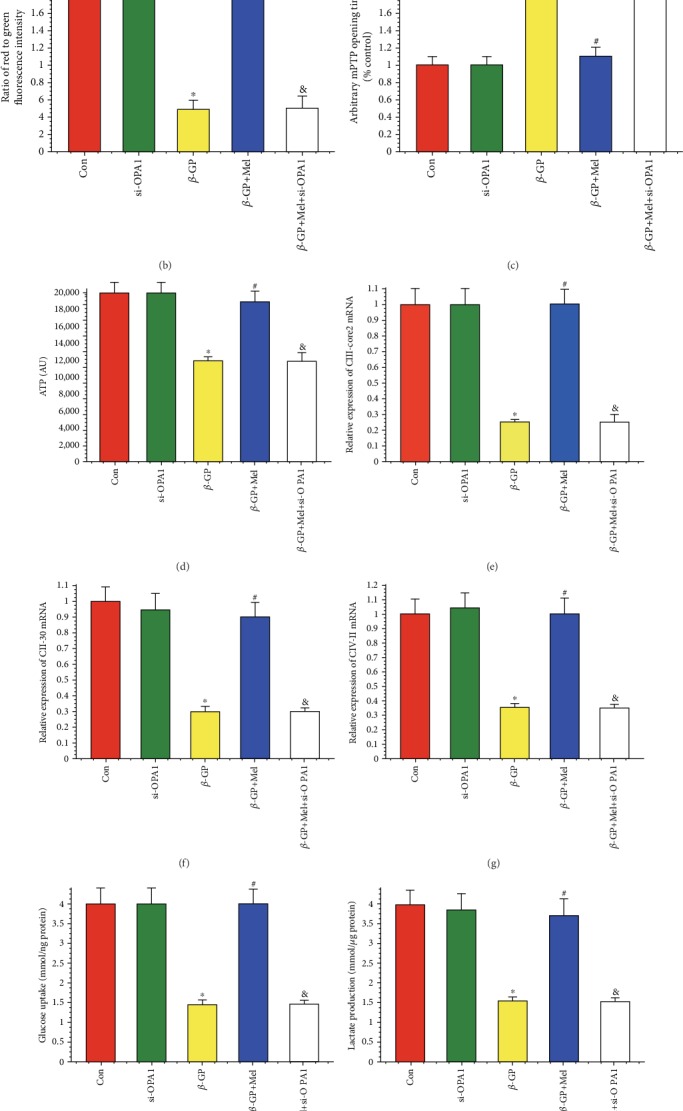
Effects of melatonin on mitochondrial membrane potential, mitochondrial permeability transition pore (mPTP) opening time, ATP production, and mitochondrial respiratory function in *β*-GP-treated VSMCs (*n* = 6/group). (a, b) The change of membrane potential (*ΔΨ*m) by JC-1 staining. (c) Result of arbitrary mPTP opening time. (d) Result of cellular ATP levels. (e–g) Results of mitochondrial respiratory complex (CIII-core2, CII-30, and CIV-II) mRNA expression. (h, i) Results of glucose uptake and lactic acid production. (j, k) Results of mitochondrial State 3/4 respiratory rate. ^∗^*P* < 0.05 vs. Con, ^#^*P* < 0.05 vs. *β*-GP, and ^&^*P* < 0.05 vs. *β*-GP+Mel.

*ΔΨ*m dissipation plays a key role in mitochondrial dysfunction. In this study, *ΔΨ*m was dissipated by *β*-GP treatment, and melatonin reversed *β*-GP-induced *ΔΨ*m dissipation via OPA1 (Figures 4(a) and 4(b)). Opening of the mPTP was promoted by treatment with *β*-GP; however, this *β*-GP-induced promotion could be attenuated by simultaneous supplementation of melatonin (Figure 4(c)). Melatonin also increased cellular ATP levels after *β*-GP treatment via OPA1 (Figure 4(d)).

Mitochondrial respiratory function was also evaluated in this study. *β*-GP decreased the mRNA expression of mitochondrial respiratory complex components (CIII-core2, CII-30, and CIV-II), which were increased when VSMCs were treated with melatonin; however, when OPA1 knockout was present, these expression levels of returned to the level of the *β*-GP group (Figures 4(e)–4(g)). Melatonin treatment significantly promoted glucose uptake and lactic acid production, but deletion of OPA1 attenuated this promotion in *β*-GP-treated VSMCs (Figures 4(h) and 4(i)). In addition, State 3/4 mitochondrial respiratory rates were found to be increased by melatonin via OPA1 regulation (Figures 4(j) and 4(k)).

### 4.5. Melatonin Maintained Mitochondrial Structural Integrity through the OPA1 Pathway

**Figure 5:**
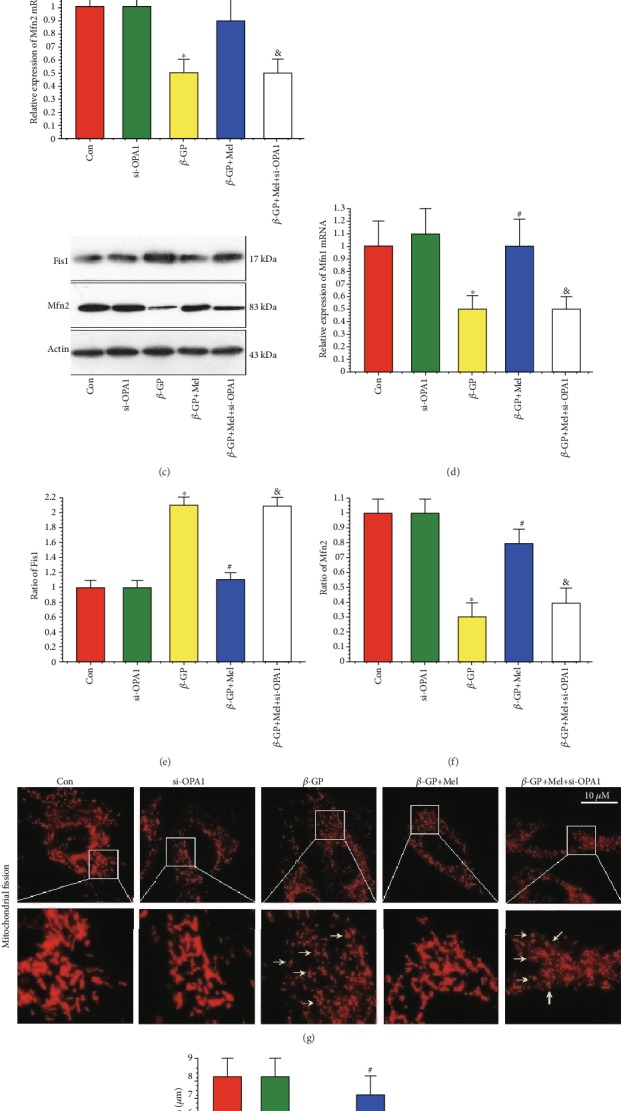
Effects of melatonin on mitochondrial fission in *β*-GP-treated VSMCs (*n* = 6/group). (a) Result of dynamin-related protein1 (Drp1) mRNA expression. (b) Result of mitochondrial fission protein 1 (Fis1) mRNA expression. (c) Result of mitofusin 2 (Mfn2) mRNA expression. (d) Result of mitofusin 1 (Mfn1) mRNA expression. (e, f) Results of Fis1 and Mfn2 protein expression. (g, h) Mitochondrial morphology was observed with the MitoTracker Red. The yellow arrows indicate the fragmented mitochondria. ^∗^*P* < 0.05 vs. Con, ^#^*P* < 0.05 vs. *β*-GP, and ^&^*P* < 0.05 vs. *β*-GP+Mel.

As shown in the qRT-PCR results, *β*-GP promoted Drp1 and Fis1 mRNA expression, and melatonin significantly inhibited *β*-GP-induced promotion. In addition, levels of Mfn1 and Mfn2 mRNA expression were significantly decreased in response to *β*-GP, and melatonin significantly increased Mfn1 and Mfn2 mRNA expression. However, these effects were negated in OPA1-deleted VSMCs. (Figures 5(a)–5(d)). This phenomenon was also confirmed by western blot analysis of Fis1 and Mfn2 (Figures 5(e) and 5(f)). Mitochondria fragmentation was significantly increased by *β*-GP, but mitochondria fragmentation was reduced by treatment with melatonin in an OPA1-dependent manner (*P* < 0.05) (Figures 5(g) and 5(h)). These results confirmed that melatonin promoted mitochondrial fusion via the OPA1 pathway.

### 4.6. Melatonin Promoted Protective Mitophagy through the OPA1 Pathway

**Figure 6:**
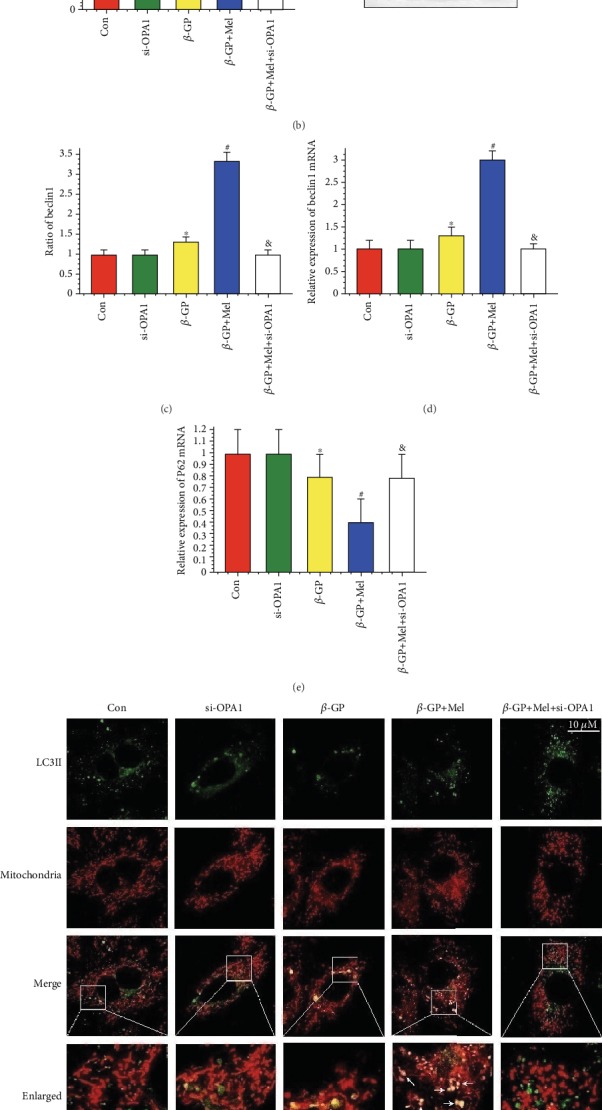
Effects of melatonin on mitophagy in *β*-GP-treated VSMCs (*n* = 6/group). (a–c) Results of LC3II/I, mito-LC3II, and beclin1 protein expression. (d) Result of beclin1 mRNA expression. (e) Result of P62 mRNA expression. (f, g) The colocation of LC3II and mitochondria in VSMCs. (h) Result of OPA1 overexpression (Ad-OPA1) or autophagy protein 5 knockout (si-ATG5) on ALP activity. ^∗^*P* < 0.05 vs. Con, ^#^*P* < 0.05 vs. *β*-GP, and ^&^*P* < 0.05 vs. *β*-GP+Mel.

Western blot results revealed that *β*-GP slightly increased mito-LC3II and beclin1 protein expression, while melatonin significantly promoted mito-LC3II and beclin1 protein expression; however, OPA1 deletion abrogated these effects (Figures 6(a)–6(c)). The effects of melatonin on beclin1 mRNA expression were consistent with western blot results (Figures 6(d) and 6(e)). Mitophagy (as indicated by an interaction between mitochondria and LC3II) was slightly upregulated by *β*-GP, and melatonin treatment significantly increased mitophagy via the OPA1 pathway (*P* < 0.05) (Figures 6(f) and 6(g)).

To investigate the effects of mitochondrial fusion/fission or mitophagy on VSMC calcification, OPA1 overexpression (Ad-OPA1), a mitochondrial division inhibitor 1 (Mdivi-1, 50 *μ*M), or siRNA against ATG5 was used in this study. Melatonin, OPA1 overexpression, or Mdivi-1 significantly reduced ALP activity in *β*-GP-induced VSMC calcification. However, the effects of melatonin were mitigated by OPA1 deletion or ATG5 knockout (Figure 6(h)). These results indicate that melatonin protected VSMCs against calcification via OPA1-related mitochondrial fusion/mitophagy.

### 4.7. Melatonin Attenuated *β*-GP-Induced VSMC Calcification through the AMPK/OPA1 Pathway

**Figure 7:**
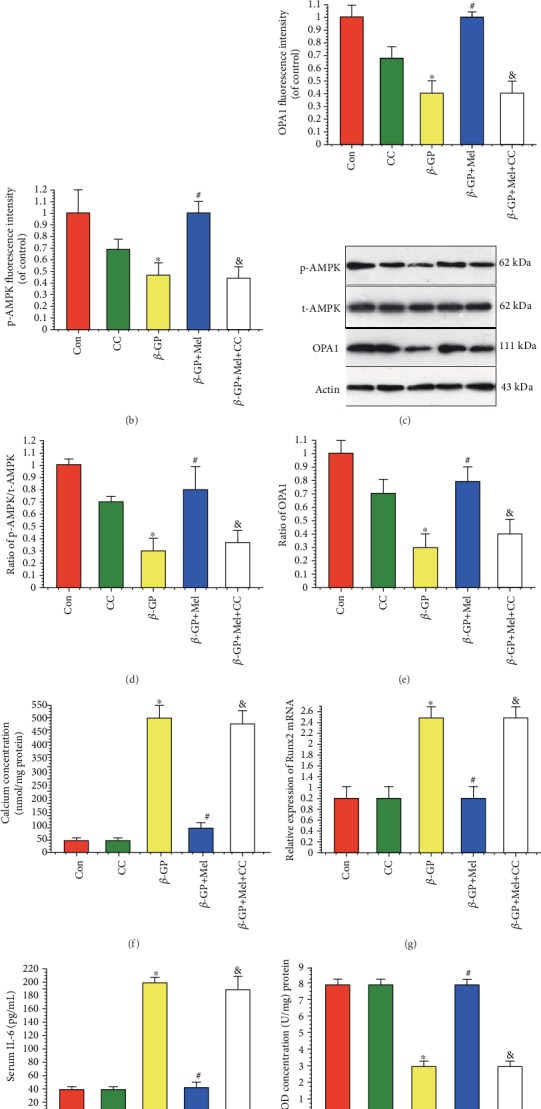
Effects of melatonin and AMPK pathway inhibitor (compound C, 1 *μ*M) on *β*-GP-induced calcification in VSMCs (*n* = 6/group). (a–c) Results of immunofluorescence assay (red signal represents p-AMPK, green signal represents OPA1). (d, e) Results of p-AMPK and OPA1 protein expression. (f) Result of calcium concentration. (g) Result of Runx2 mRNA expression. (h) Result of interleukin-6 (IL-6) level. (i) Result of SOD level. (j) Result of caspase 3 activity. ^∗^*P* < 0.05 vs. Con, ^#^*P* < 0.05 vs. *β*-GP, and ^&^*P* < 0.05 vs. *β*-GP+Mel.

Immunofluorescence staining results showed that melatonin treatment increased p-AMPK and OPA1 expression in calcifying VSMCs. We also found that compound C could inhibit the effect of melatonin on OPA1, as indicated by decreased OPA1 and p-AMPK signals in the compound C-treated group compared with the melatonin group (Figures 7(a)–7(c)). This result was supported by western blot analysis (Figures 7(d) and 7(e)). Melatonin significantly reduced calcium deposition, Runx2 mRNA expression, interleukin-6 level, and caspase 3 activity in *β*-GP-induced calcified VSMCs (Figures 7(f)–7(j)). However, compound C reduced the protective effects of melatonin on VSMC calcification. Overall, these results indicate that melatonin attenuated *β*-GP-induced VSMC calcification via the AMPK/OPA1 signaling pathway.

## 5. Discussion

In the present study, we investigated the effects of melatonin on VSMC calcification and the molecular mechanism by which these effects were produced. Our results suggest that the observed decrease of VSMC calcification induced by melatonin was mediated, at least in part, by AMPK/OPA1 signaling.

The effect of melatonin on calcification has recently been investigated [20–22]. Son et al. found that melatonin could promote osteoblastic differentiation and mineralization of preosteoblastic MC3T3-E1 cells under hypoxic conditions [20]. However, Kumar et al. showed that melatonin significantly antagonized cyclosporine-induced renal impairment. Microcalcification of the corticomedullary junction subsequent to cyclosporine administration was prevented by melatonin [21]. Zhang et al. demonstrated that melatonin treatment suppresses oxidative stress-induced calcification and apoptosis in endplate chondrocytes [22].

Mitochondrial fusion and mitophagy have been implicated in vascular calcification. A study by Kim et al. observed the disruption of mitochondrial structural integrity in calcifying VSMCs. However, *α*-lipoic acid inhibited VSMC apoptosis and calcification by promoting mitochondrial fusion [23]. Ma et al. found that mitochondrial mass was decreased after *β*-GP exposure, whereas metformin treatment increased mitochondrial fusion compared with the *β*-GP group. Metformin attenuated *β*-GP-induced vascular calcification by enhancing mitophagy. Inhibition of autophagy by small interfering RNA targeting ATG5 aggravated *β*-GP-induced calcification [6].

OPA1 has been shown to inhibit reperfusion injury in the heart and brain by promoting mitochondrial fusion. Wei et al. showed that melatonin increased OPA1 expression, enhanced mitochondrial fusion, and reduced neuron death during brain reperfusion injury. These protective effects of melatonin were negated by OPA1 knockout [24]. The findings of Ma and Dong suggest that melatonin attenuated cardiac ischemia reperfusion injury by upregulating OPA1-related mitochondrial fusion, whereas OPA1 depletion abolished the protective effects of melatonin on cardiac reperfusion injury [25]. Zhang et al. found that melatonin attenuated cardiac reperfusion injury by activating OPA1-related mitochondrial fusion and mitophagy; however, ATG5 deletion abolished these protective effects [9]. Our experiments demonstrated that melatonin increased OPA1-related mitochondrial fusion/mitophagy and reduced VSMC calcification. However, OPA1 knockout reduced the effects of melatonin on mitochondrial fusion/mitophagy and inhibited the effects of melatonin on VSMC calcification. Our results suggest that melatonin inhibits VSMC calcification through OPA1-related mitochondrial fusion/mitophagy.

**Figure 8:**
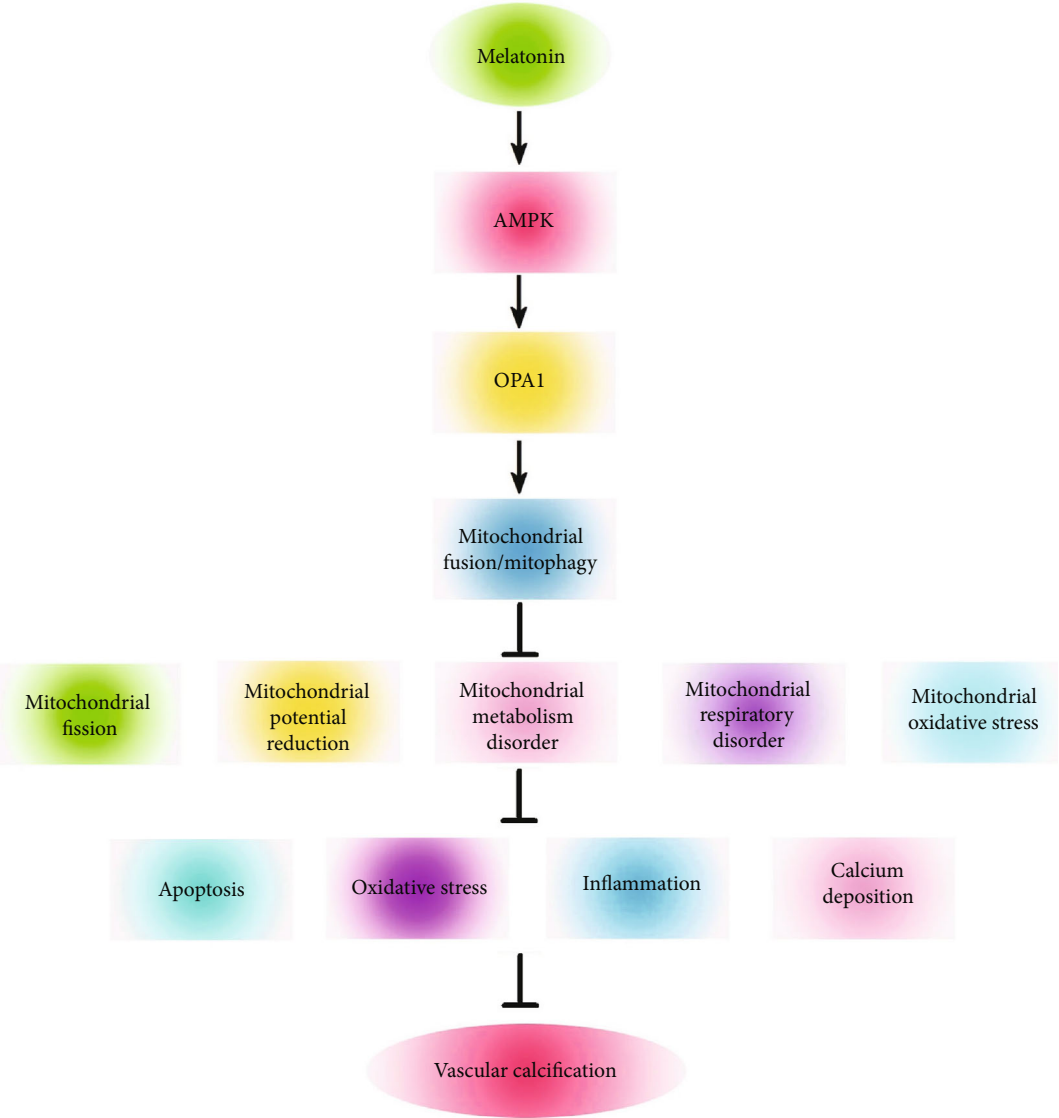
Schematic representation showing that melatonin regulates VSMC calcification through an AMPK/OPA1 signaling pathway. Melatonin activated AMPK expression, which in turn enhanced OPA1 and, subsequently, promoted mitochondrial fusion/mitophagy. Activation of mitochondrial fusion/mitophagy reduced apoptosis, oxidative stress, inflammation, and calcium deposition. These effects subsequently inhibited VSMC calcification.

Mitochondrial fusion/mitophagy is related to AMPK, a key energy sensor that regulates cellular metabolism to maintain energy homeostasis [26, 27]. Cui et al. showed that melatonin treatment reduced the apoptosis of human umbilical vein endothelial cells by promoting mitochondrial fusion through activation of the AMPK pathway [13]. Zhang et al. found that melatonin increased OPA1 expression via the AMPK pathway, whereas compound C reduced OPA1 expression and negated the effects of melatonin [9]. In this study, melatonin activated AMPK protein expression, promoted OPA1-required mitochondrial fusion/mitophagy, and reduced VSMC calcification. Furthermore, we used an AMPK inhibitor to evaluate the effects of melatonin on VSMC calcification and found that compound C reduced the effects of melatonin on AMPK/OPA1 and increased VSMC calcification. Taken together, melatonin activated AMPK expression, which in turn enhanced OPA1 to subsequently promote mitochondrial fusion/mitophagy. Activation of mitochondrial fusion/mitophagy reduced apoptosis, oxidative stress, inflammation, and calcium deposition. These effects subsequently inhibited VSMC calcification (Figure 8).

There are a few limitations to our study. First, the findings are only based on in vitro experiments. Second, as we only observed caspase 3 activation in the study, other apoptosis signals should be evaluated in the future. Third, an AMPK knockdown system may better validate the role of AMPK in melatonin-reversed VSMC calcification induced by *β*-GP. More research is needed to further clarify the mechanism of melatonin on vascular calcification.

## 6. Conclusions

Our study demonstrated that melatonin played an important and protective role in VSMCs by inhibiting calcification via the AMPK/OPA1 system.

## Figures and Tables

**Table 1:** Real-time qPCR primers.

Gene	Forward primer (5′->3′)	Reverse primer (5′->3′)
MMP9	AATCTCTTCTAGAGACTGGGAAGGAG	AGCTGATTGACTAAAGTAGCTGGA
MIP1*α*	CTGCCCTTGCTGTCCTCCTCTG	CTGCCGGCTTCGCTTGGTTA
IL-8	TCCTAGTTTGATACTCCCAGTC	ACAAGTTTCAACCAGCAAGA
CII-30	CCTCTAGATACCGATAGCC	AACTTACGATAGGCTGATCCG
CIII-core2	CTCTAGGAATCCGATAGTCTA	GGCAAGTAGATACCAGTA
CIV-II	GAGGGTCCTAGATCCGAT	GTTGACCAGACCATAGTCCAT
Drp1	AGACCTCTCATTCTGCAACTG	TTACCCCATTCTTCTGCTTCC
Fis1	TGTCCAGTCCGTAACTGAC	TTCGATACCTGACTTAC
Mfn2	ATGTGGCCCAACTCTAAGTG	CACAAACACATCAGCATCCAG
Mfn1	ACCTGTTTCTCCACTGAAGC	TGGCTATTCGATCAAGTTCCG
Beclin1	GGCAGTGGCTCCTATT	GGCGTGCTGTGCTCTGAAAA
P62	CTCGCTATGGCGTCGCTCACCGTG	TCACTCTGGCGGGAGATGTGGGTA
Runx2	CACTGGCGGTGCAACAAGA	TTTCATAACAGCGGAGGCATTTC
*β*-Actin	GATGGTGGGTATGGGTCAGAAGGAC	GCTCATTGCCGATAGTGATGACT

## Data Availability

The data used to support the findings of this study are available from the corresponding author upon request.
